# Effect of age-related hyperkyphosis on depression and quality of life

**DOI:** 10.22088/cjim.14.1.31

**Published:** 2023

**Authors:** Gulcimen Soylu, Guzin Cakmak, Yusuf Yalvac, Zeynel Abidin Öztürk

**Affiliations:** 1 Sanliurfa Eyyubiye Training and Research Hospital, Department of Internal Medicine, Division of Geriatric Medicine, 63250 Eyyubiye, Sanliurfa, Turkey; 2 Gaziantep University, Faculty of Medicine, Department of Internal Medicine, Division of Geriatric Medicine, 27100 Sahinbey, Gaziantep, Turkey; 3 Inonu University, Faculty of Medicine, Department of Internal Medicine, 44210 Battalgazi, Malatya, Turkey

**Keywords:** Age-related hyperkyphosis, Bloc method, Cobb angle, Chronic pain, Depression, Quality of life

## Abstract

**Background:**

Hyperkyphosis is a frequent problem in older adults. Depressed mood and decreased quality of life are supposed to be related to age-related hyperkyphosis. This study aimed to explain the relation between depression, quality of life, and hyperkyphosis in old patients.

**Methods:**

142 patients who applied to the outpatient clinic of geriatrics were enrolled in this cross-sectional study. Mean age of participants was 72. Hyperkyphosis was evaluated by the bloc method defined in the Rancho Bernardo Study (1). Depression was evaluated by the Short form of Yesavage Geriatric Depression Scale (GDS). Quality of life was assessed by the 3-level version of EQ-5D.

**Results:**

Hyperkyphosis was found to be positively related to depression (P=0.037), negatively related to the QOL (p<0.001). QOL, depression, and hyperkyphosis were in a ship with each other when evaluated with one-way MANOVA (F [3.135] =5.23, P=0.002, Wilk's Λ=0.896, partial η2=0.104). Chronic pain was negatively related to QOL (p<0.001). Depression was positively related to chronic pain (p<0.001). QOL evaluated with VAS was independently related to the presence of hyperkyphosis in logistic regression analysis (r2=0.179, P=0.007).

**Conclusion:**

Considering the relationship with depression and quality of life, early recognition, and treatment of hyperkyphosis in elderly individuals is important. More studies evaluating the association between postural disorders, quality of life and mood disorders in older adults will be useful.

Hyperkyphosis which can be considered as a new geriatric syndrome is common in older adults and is thought to be associated with mood and quality of life. Increased forward curvature of the thoracic vertebra that increases in old age is defined as age-related hyperkyphosis(2). The incidence of hyperkyphosis in people over 60 years old is between 20% and 40% (3). Hyperkyphosis can adversely affect pulmonary function and daily living activities, impairs quality of life, and increases mortality (4). Depressive syndromes often affect older adults with co-morbidities (5). It was previously asserted by different researchers that posture may be related to the mood in older adults. Youkyung et al. revealed a relationship between posture and depressive symptoms in patients with Parkinson's disease (6). Quality of life (QOL) in the elderly is an issue that is affected by many parameters and there are many studies on this subject. Spinal deformities and posture were shown to be related to QOL (7). Depression is a condition that affects the quality of life both directly and through its effect on other factors (8). 

Chronic pain is a condition that occurs in older ages and causes loss of functional capacity (9). Chronic pain seen in older adults can be caused by many reasons like age-related hyperkyphosis and other spinal deformities (10). It can be argued that chronic pain is also related to mood and quality of life. Considering this information, we planned a study evaluating the relation between age-related hyperkyphosis, depression, and quality of life. We also aimed to evaluate their relation with chronic pain.

## Methods

**Patients and study design: **We organized a cross-sectional study to evaluate the relations between hyperkyphosis, depression, and quality of life. Seventy-one hyperkyphotic and seventy-one normal patients who applied to the polyclinic of geriatrics with other complaints between January 2017 and January 2018 were included in the study. Those who were hyperkyphotic were included in the study group and those who were not hyperkyphotic were in the control group with an experienced staff. Measurements, tests, and data recording were carried out by the other staff. Additionally, each task was done by the same person during the study. Patients who were under 65 years of age or suffering from acute or chronic infections and dementia were excluded from the study. Osteoporotic patients, corticosteroid users, tobacco users, and alcohol users were also excluded. The Gaziantep University Local Research Ethics Committee approved this study with reference number 2017/18. Informed consent were obtained from the participants. 

**Figure 1:**
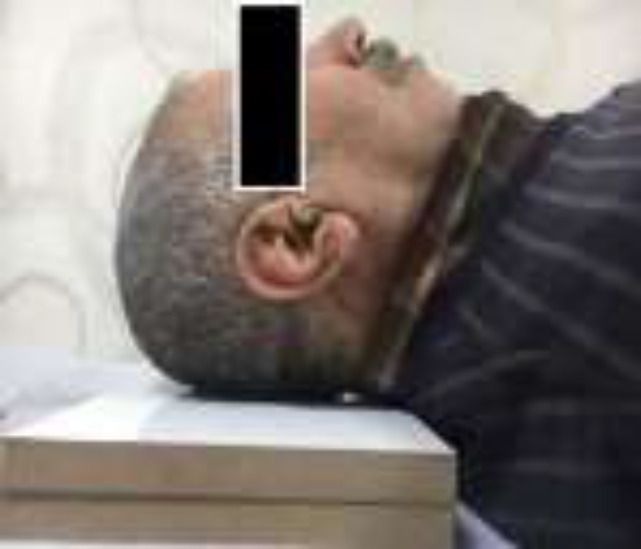
Measurement of hyperkyphosis by bloc method Patients whose neutral position required to align the eyes to the ceiling could be achieved with the support of one or more blocks were defined as hyperkyphotic

**Measurement of hyperkyphosis: **Kyphosis was assessed by the bloc method in the Rancho Bernardo Study (1). When evaluating hyperkyphosis with the bloc method, patients were brought to the radiology table. Blocks that 1.7 cm in height were placed under the heads of patients to keep them in a neutral position and their eyes were directed to the ceiling. Neutral position can be described as the absence of both hyperflexion and hyperextension. Numbers of the blocks used for keeping patients in neutral position were recorded. In this study, hyperkyphosis was the state in one or more blocks needed to restore a neutral position. The implementation of the block method is summarized in figure 1.

**Assessment of depressive status: **The Yesavage Geriatric Depression Scale (GDS)-Short form is used to evaluate patients for depression. Scores of 5 and above were accepted as valid for depression (11).

**Assessment of dementia: **The standardized form of mini-mental state examination (MMSE) was used to exclude dementia diagnosis in patients. The MMSE patients were tested in about six different areas; orientation, registration, attention, calculation, language, and recall (12). Scores of ≤24 were valid for the presence of dementia.

**Assessment of life quality:** Quality of life was evaluated by the 3-level version of EQ-5D (EQ-5D-3L), which was introduced in 1990 by the EuroQol Group. The EQ-5D-3L mainly consists of two parts: the EQ-5D descriptive system and the EQ visual analog scale (EQ-VAS). The descriptive system evaluates the patients for five dimensions: mobility, self-care, usual activities, pain/discomfort, and anxiety/depression. There are three levels in each dimension (no problems, some problems, and extreme problems), and patients mark the level that is right for them. This selection results in a 1-digit number that expresses the level selected for that dimension. The digits for the five dimensions can be combined into a 5-digit number that shows the health status of the patient. In EQ-VAS, patients give a score out of 100 according to their perception of their health status (13).

**Assesment of chronic pain: **The pain status of the patients was evaluated with a visual analog scale (VAS). Patients gave themselves a score between 0 and 100 for their pain status(14).

**Data analysis:** The variables were analyzed for their distribution normality using the Kolmogorov–Smirnov and Shapiro Wilk test. All data were disturbed normally (p>0.05). Descriptive statistics are given for continuous variables. Continuous variables of groups were assessed by using the independent sample t-test. The data were expressed as mean ± deviation (S.D.). Relationships between parameters were investigated by the chi-square test, Pearson correlation analysis, and multivariate analysis of variance (MANOVA). We used logistic regression to simulate a model to determine factors affecting hyperkyphosis. We used SPSS Version 22.0 (IBM, Armonk, NY) to analyze the data.

## Results

Patients who were admitted to Gaziantep University Hospital Outpatient Polyclinic of Geriatrics between January 2017 and January 2018 were evaluated. There were 142 patients, 36 (25%) of them were males, and 106 (75%) of them were females. The mean age of participants was 72. Fifty-two percent of female patients and 44% of male patients were hyperkyphotic. There was no statistical significance with gender (P=0.189). 

Hyperkyphosis was found to be positively related to depression by correlation analysis (P=0.037, r=0.178). 55% of patients with hyperkyphosis and 45% of those without hyperkyphosis were depressive. It was observed that hyperkyphosis was negatively correlated with the QOL assessed by the descriptive system (P=0.013, r=-0.207), and QOL assessed by VAS (p<0.001, r=-0.492). There was a significant relationship between QOL (both evaluated with descriptive system and VAS), depression, and hyperkyphosis when evaluated with one-way MANOVA (F [3.135] =5.23, P=0.002, Wilk's Λ=0.896, partial η2=0.104). All dependent variables were revealed to be related to hyperkyphosis in the post-hoc analysis (P=0.015, p<0.001, P=0.036). Results of MANOVA were summarized in table 2. It was revealed that the QOL assessed by the descriptive system was negatively correlated with chronic pain (p<0.001, r=-0.457). The QOL assessed by VAS was negatively correlated with chronic pain too (p<0.001, r=-0.329). Depression was positively related to chronic pain (p<0.001, r=0.377). The results of quality of life, depression, and chronic pain assessments in patients with and without hyperkyphosis are summarized in table 1.

We found that QOL evaluated with VAS was independently related to hyperkyphosis in logistic regression analysis (r²=0.161, P=0.011). Results of logistic regression analysis were summarized in table 3.

**Table 1:** Relationship between the presence of hyperkyphosis and other parameters

	**Hyperkyphotic (n=71)**	**Non-hyperkyphotic (n=71)**	**P=**
Age	73.8 ± 9.02	70.5 ± 6.4	0.013*
3-level version of EQ-5D (descriptive)	0.87 ± 0.21	0.78 ± 0.21	0.013*
3-level version of EQ-5D (VAS)	52.35 ± 18	63.71 ± 16.9	<0.001*
GDS-SF	6.3 ± 4.4	4.57 ± 5.1	0.037*
Chronic pain VAS	44.1 ± 27	42 ± 22.5	0.62

**VAS: **visual analog scale

**GDS-SF:** geriatric depression scale-short form

**Table 2:** Results of MANOVA

**Dependent** **Variables**	**Type III** **Sum of Squares**	**Df**	**Mean Square**	**F**	**P**
3-level version of EQ-5D (descriptive)	0.275	1	0.275	6.131	0.015*
3-level version of EQ-5D (VAS)	4489.343	1	4489.343	14.764	<0.001*
GDS-SF	102.611	1	102.611	4.481	0.036

** VAS: **Visual analog scale

**GDS-SF:** Geriatric depression scale-short form

**Table 3:** Logistic regression analysis results

**Variables**	**B**	**S.E.**	**Wald**	**Exp(B)**	**P**
3-level version of EQ-5D (descriptive)	0.310	1.013	0.093	1.363	0.760
3-level version of EQ-5D (VAS)	-0.031	0.012	6.489	0.969	0.011*
Age	0.042	0.025	2.724	1.043	0.099
GDS-SF	0.024	0.043	0.298	1.024	0.585

** VAS: **Visual analog scale

**GDS-SF:** Geriatric depression scale-short form

**S.E.: **Standard error

## Discussion

In this study, we found a significant relation between the presence of age-related hyperkyphosis, depression and life quality in older adults. Studies analyzing the relation between mood and spine problems have been conducted before. Smith et al. evaluated the effect of preoperative depression on 2-year clinical outcomes following adult spinal deformity surgery. They revealed that a baseline clinical history of depression did not correlate with worse 2-year outcomes after adult spinal deformity surgery after adjusting for baseline differences in QOL, comorbidities, and severity of the spinal deformity (15). Toombs et al. also designed a study to evaluate the effect of psychological disturbances on adult spinal deformity surgery outcomes, they did not find any significant effect. They revealed that the rate of comorbid psychiatric diagnoses in patients with adult spinal deformity was significantly higher than in patients with adult spinal fusion. The frequency of mood disorders in these two groups was 9.6% vs 6.9% (16). These rates are extremely low compared to our study. We can attribute this difference to the age difference between study populations. 

Unlike these studies, we evaluated whether hyperkyphosis influenced the presence of depression by making a comparison with a control group. Our study also differs from this study that was conducted only with older adults. Gallant et al. published a review evaluating the effect of idiopathic scoliosis on body image, eating behavior, and mood disorders in adolescents. They concluded that in adolescent idiopathic scoliosis, body perception changes, fear of gaining weight, and related eating disorders increase, and mood disorders are more common. This study is similar to our study in terms of showing the effect of posture deformities on mood (17). Moslehi et al. also revealed a relationship between kyphosis, anxiety, depression, and aggression in high school boy students (18).

In our study, a significant relation was also found between hyperkyphosis and decreased quality of life. In a systematic review published by Anwer et al., it was concluded that exercise improves the life quality in patients with adolescent idiopathic scoliosis(19). Aboutorabi et al. revealed in their systematic review and meta-analysis that spinal orthoses and taping positively affect the quality of life in the elderly with thoracic hyperkyphosis (18). Sangtarash et al. demonstrated that there is a negative correlation between the kyphotic index and quality of life in osteoporotic women with hyperkyphosis(20). This study differs from our study in that it was conducted only in women and patients who had spinal osteoporosis aged 50-68. We included only male and female patients over 65 years of age and without osteoporosis in our study. It has been shown in previous studies that hyperkyphosis can occur without osteoporosis and this is mostly related to lumbar disc degeneration (21).

In our study, we concluded that chronic pain was positively correlated with depression and negatively correlated with quality of life. Burke et al. concluded that high-intensity pain and neuropathic pain negatively affect the quality of life after spinal cord injury (22). Husky et al. revealed that chronic back pain was related to lower scores on all SF-36 mean scores and on the Physical Composite Score and Mental Composite Score(23). Imagama et al. concluded that positive spinal curvature and chronic pain were associated with the reduced physical quality of life (24). Zis et al. stated that depression and chronic pain coexist in 13% of the elderly population in a review they published (25). Stubbs et al. conducted a meta-analysis study to explain the relationship between depression and pain in low- and middle-income countries. Depression was found to be significantly associated with severe pain in 44/47 countries with a pooled odds ratio of 3.93 (95% CI 3.54–4.37) (26). Our study is valuable and unique evaluating the relationship between age-related hyperkyphosis, depression, and quality of life at the same time. It is also an interesting finding that the use of visual analog scale in quality-of-life assessment reveals the relation with hyperkyphosis better than the descriptive scale.

### Limitations:

However, our study had some limitations. Seventy-five percent of our study population were females. It could be more accurate to evaluate this issue in a gender-balanced population. In our study, while we were questioning chronic pain, we questioned pain in any part of the body. It could be better to explain this relationship in terms of pain specific to the area. Prospective studies with larger populations that evaluate the effect of treatment of hyperkyphosis on depression and quality of life could be beneficial.

In conclusion, we found a relation between depression, quality of life, and age-related hyperkyphosis. Early diagnosis and treatment of hyperkyphosis, which is an important health problem for the elderly, is important for preventing depression and increasing the quality of life. More studies should be done on this issue. 
